# SaGP: identifying plant saline-alkali tolerance genes based on machine learning techniques

**DOI:** 10.3389/fpls.2025.1629794

**Published:** 2025-07-16

**Authors:** Baixue Qiao, Wentao Gao, Xudong Zhang, Min Du, Shuda Wang, Xuanrui Liu, Shaozi Pang, Chunxue Yang, Jiang Wang, Yuming Zhao, Linan Xie

**Affiliations:** ^1^ School of Ecology, Northeast Forestry University, Harbin, China; ^2^ Key Laboratory of Saline-Alkali Vegetation Ecology Restoration, Ministry of Education, Northeast Forestry University, Harbin, China; ^3^ State Key Laboratory of Tree Genetics and Breeding, Northeast Forestry University, Harbin, China; ^4^ College of Computer and Control Engineering, Northeast Forestry University, Harbin, China; ^5^ College of Landscape Architecture, Northeast Forestry University, Harbin, China; ^6^ College of Life Science, Northeast Forestry University, Harbin, China; ^7^ Key Laboratory of Sustainable Forest Ecosystem Management-Ministry of Education, School of Ecology, Northeast Forestry University, Harbin, China

**Keywords:** machine learning, saline-alkali tolerance genes, gene mining, feature selection, SAGP

## Abstract

Mining novel genes underlying agronomical traits is a crucial subject in plant biology, essential for enhancing crop quality, ensuring food security, and preserving biodiversity. Wet experiments are the main methods to uncover genes with target functions but are expensive and time-consuming. Machine learning, in contrast, can accelerate the gene discovery process by learning from accumulated data, making it more efficient and cost-effective. However, despite their potential, existing machine-learning tools to mine stress-resistant genes in plants are scarce. In this study, we developed the first known machine learning model, SaGP (Saline-alkali Genes Prediction), to identify plant saline-alkali tolerance genes based on sequencing data. It outperformed traditional computational tools, *i.e.*, BLAST, and correctly identified the latest published genes. Moreover, we utilized SaGP to evaluate three recently published genes: *GhAG2*, *MdBPR6*, and *TaCCD1*. SaGP correctly identified all their functions. Overall, these results suggest that SaGP can be used for the large-scale identification of saline-alkali tolerance genes and served as a framework for the development of additional automated tools, thus promoting crop breeding and plant conservation. To efficiently identify salt-alkali resistant genes in large-scale data, we developed a user-friendly, freely accessible web service platform based on SaGP (https://www.sagprediction.com/).

## 1 Introduction

Enhancing plants’ tolerance to abiotic stresses has long been the focus in biology and breeding science. Early efforts focused purely on plant phenotypes (Meuwissen et al., 2001; Meyer et al., 2012). Later works began to decipher the genetic bases underlying key traits based on quantitative trait loci (QTL) mapping (Kang et al., 2019) and genome-wide association studies (GWAS) (Gupta, 2021). Many functional genetic variants have been identified, resulting in breeding plants with excellent traits (Wang et al., 2016; Zhao et al., 2024) and developing effective species conservation strategies (Chen et al., 2022; Gougherty et al., 2021). However, despite these achievements, these works are time-consuming and costly (Dou et al., 2021) and overall have low precision in determining functional variants (Mackay et al., 2009; Wray et al., 2013), resulting in inefficient plant selection and breeding. Moreover, they focus on a few model species, such as Arabidopsis, maize, rice, etc. Taking full advantage of knowledge from these species and utilizing them to boost the identification of functional genetic variants in other species are still challenging.

With the development of genetics and informatics, a new framework is now being proposed to boost efficiency and cut the cost of current research, i.e., Breeding 4.0 (Wallace et al., 2018). It is characterized by high-throughput sequence data (Ding et al., 2023; Yang et al., 2013) combined with computational methods (Jarrahi, 2018). Traditional computational methods, such as BLAST (Altschul et al., 1990), may fit Breeding 4.0, but their poor accuracy can lead to inefficiency (Dai et al., 2020; Li et al., 2022). On the other hand, machine learning (ML) may provide an alternative to traditional computational approaches (Fu et al., 2023; Qiao et al., 2024; Van Dijk et al., 2021). It has been used in genomic selection-assisted breeding (Yan and Wang, 2023) and in assessing plants’ vulnerability under future climates regarding their genetic compositions (Sang et al., 2022). Moreover, several studies have implemented machine learning algorithms to identify plant genes with specific functions. For example, PGB was used to detect photosynthetic-related genes based on a voting algorithm (Wang et al., 2022). DRPPP based on SVM was created to predict disease resistance proteins with high performances (Pal et al., 2016). ConSReg based on regularized LASSO was developed to identify key transcription factors responsive to specific abiotic stresses, which outperformed traditional enrichment-based methods (Song et al., 2020). These works can provide important tools for Breeding 4.0 to precisely screen target genes on a large scale and thus facilitate crop improvement and species conservation. Unfortunately, similar work to identify genes resistant to abiotic stresses are scarce.

In this study, we proposed a framework to construct intelligent tools to identify novel plant abiotic stress resistant genes. Focusing on saline-alkali stress, i.e., excessive accumulation of neutral salts and sodic salts that leads to decreased crop productivity (James et al., 2012) and the loss of native biodiversity worldwide (Briggs and Taws, 2003), we developed the first known machine learning model (SaGP, Saline-alkali Genes Prediction) to identify plant saline-alkali tolerance genes. It achieves 0.99 prediction accuracy better than BLAST assessed with the independent test dataset. To further evaluate the performance of SaGP, we tested some latest published genes, including *GhAG2* (Yu et al., 2022), *MdBPR6* (Zhang et al., 2023), and *TaCCD1* (Cui et al., 2023), and SaGP correctly identified all their functions. Overall, the results suggest that SaGP can be used to fast and accurately identify saline-alkali tolerance genes in plants on a large scale with sequencing data, thus promoting crop breeding and plant conservation. SaGP is freely available at www.sagprediction.com.

## 2 Results

### 2.1 Model comparison and SaGP construction

**Figure 1:**
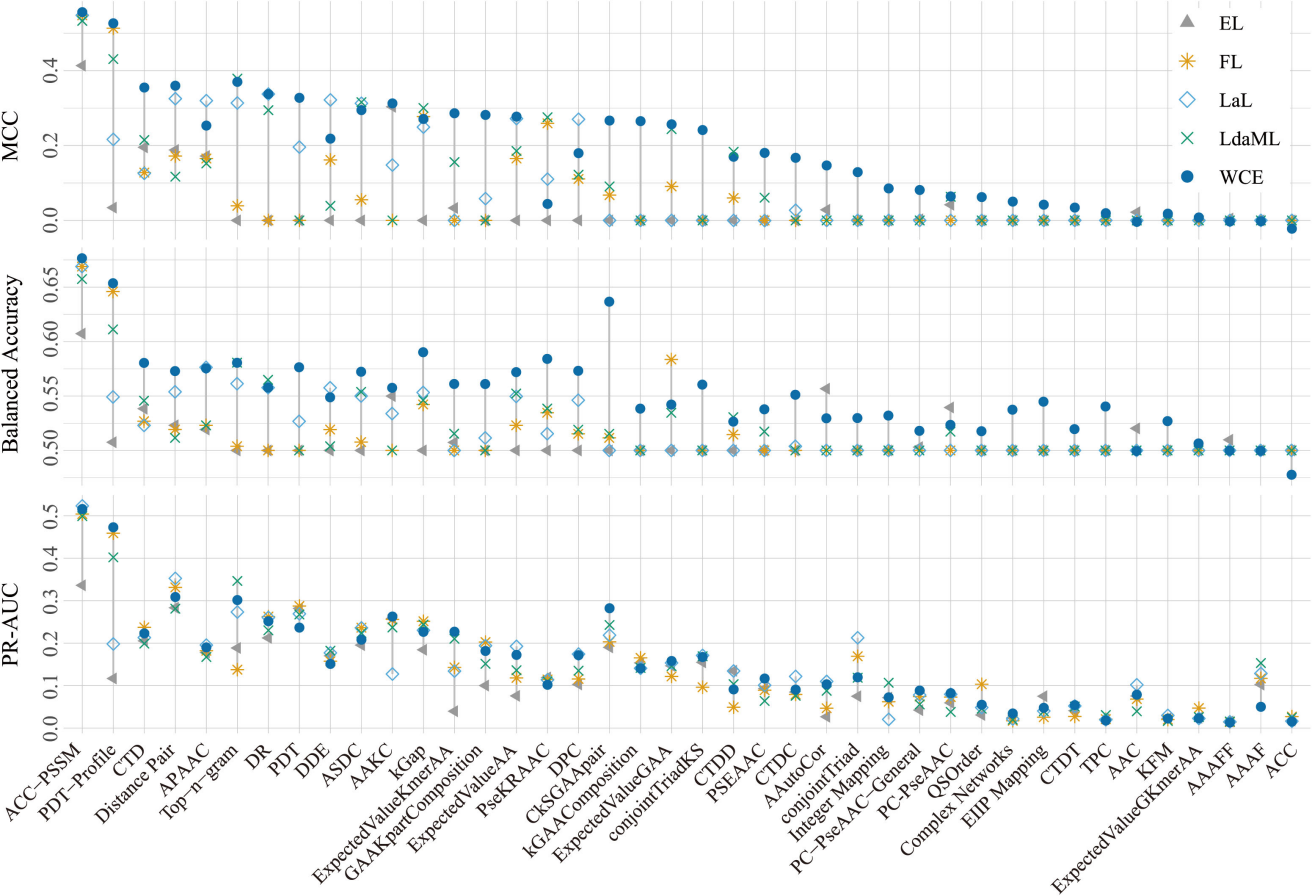
The performances of five cost-sensitive methods and 40 groups of protein features based on the test dataset. EL, Equalization loss; FL, Focal loss; LaL, Logit-adjusted loss; LdaML, Label-distribution-aware margin loss; WCE, weighted cross-entropy; MCC, Matthew’s Correlation Coefficient; PR-AUC, the area under the precision-recall. See **Table 3** for the details of 40 groups of protein features.

**Figure 2:**
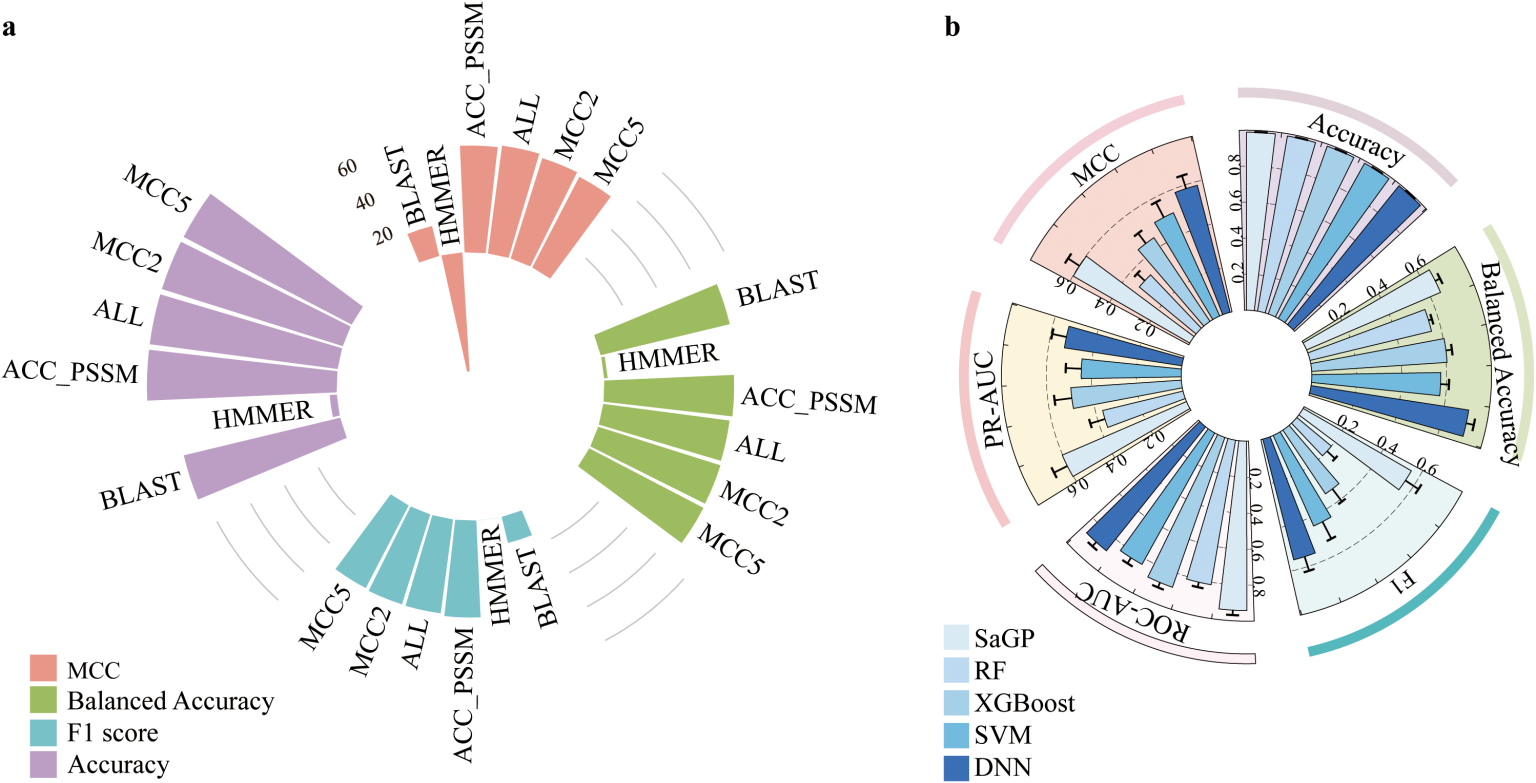
**(a)** The performances of ACC-PSSM, all features, MCC2, MCC5, BLAST and HMMER based on the test dataset. **(b)** Performance comparison of SVM, RF, XGBoost, DNN, and SaGP on the independent test dataset.

The five cost-sensitive methods performed differently regarding their capacity to distinguish saline-alkali tolerance and non-tolerance genes. Overall, the Weighted Cross-Entropy (WCE) method had the best performances regarding MCC, Balanced Accuracy, and PR-AUC (**Figure 1**; see **Supplementary Figure S1** for Accuracy, F1 score, and ROC-AUC values). Moreover, different groups of features showed different pertinency to the gene function of saline-alkali tolerance. Among them, ACC-PSSM achieved the highest and most stable performances across all metrics, followed by PDT-Profile (**Figure 1**; **Supplementary Figure S1**). In contrast, several features, such as ACC and AAAFF, had the lowest performances (**Figure 1**; **Supplementary Figure S1**). We further compared the performances of SaGP models constructed using four different feature sets—ACC-PSSM, all features, and the top two and top five groups ranked by MCC—with those of traditional tools HMMER and BLAST. The performances of BLAST were generally low with respect to MCC, Balanced Accuarcy, F1 score, and Accuracy (**Figure 2a**). Only 84% and 3.6% of saline-alkali tolerance genes can be correctly identified by BLAST (**Figure 2a**), respectively, suggesting their inability to screen for saline-alkali tolerance genes on a large scale. The set with all features performed slightly better than ACC-PSSM in terms of MCC, while ACC-PSSM performed best regarding F1 score and Balanced Accuracy (**Figure 2a**). Because extracting all features is time-consuming, we thus implemented SaGP based on ACC-PSSM.

Next, we compared the classification performances of the SaGP with the other four classifiers—SVM, Random Forest (RF), XGBoost, and deep neural network (DNN)—under the same cost-sensitive learning setting using the WCE loss function. The comparison was based on five evaluation metrics: Accuracy, F1 score, Area Under the ROC Curve (AUROC), Area Under the Precision-Recall Curve (AUPRC), and MCC. Among all models, SaGP outperformed all other classifiers, achieving the highest MCC (0.5988) and AUPRC (0.6021) (**Table 1**, **Figure 2b**), which underscores its superior ability to correctly identify saline-alkali tolerance genes under imbalanced conditions. It also attained competitive values in F1 score (0.5563) and AUROC (0.9408) (**Table 1**, **Figure 2b**), indicating both reliable classification and strong ranking capability.

**Table 1:** Performance of SVM, RF, XGBoost, DNN, and SaGP on the independent test dataset.

Model	Accuracy	Balanced Accuracy	F1	ROC-AUC	PR-AUC	MCC
SaGP	0.989 ± 0.0006	0.649 ± 0.0226	0.556 ± 0.0688	0.941 ± 0.0261	0.602 ± 0.0651	0.598 ± 0.0600
RF	0.987 ± 0.0005	0.556 ± 0.0147	0.200 ± 0.0473	0.814 ± 0.0265	0.368 ± 0.0669	0.328 ± 0.0452
XGBoost	0.988 ± 0.0007	0.604 ± 0.0256	0.341 ± 0.0694	0.882 ± 0.0281	0.493 ± 0.0762	0.448 ± 0.0558
SVM	0.987 ± 0.0012	0.574 ± 0.0361	0.441 ± 0.0842	0.835 ± 0.0351	0.445 ± 0.0683	0.503 ± 0.0750
DNN	0.99 ± 0.0011	0.710 ± 0.0333	0.554 ± 0.0645	0.883 ± 0.0300	0.529 ± 0.0632	0.583 ± 0.0592

To further evaluate the capacity of SaGP to identify novel saline-alkali tolerance genes, we predicted the three latest published genes, i.e., *GhAG2* (Yu et al., 2022), *MdBPR6* (Zhang et al., 2023), and *TaCCD1* (Cui et al., 2023). The predictions were consistent with the experimental results in the literature (**Table 2**), supporting that SaGP can correctly uncover novel saline-alkali tolerance genes.

**Table 2:** The 40 groups of protein features extracted in our study, their abbreviations, and corresponding tools.

Gene	SaGP Prediction	Experiment	Description
*GhAG2*	yes	salt resistance	In cotton, the over-expression of *GhAG2* increased the germination rate under the saline environment (Yu et al., 2022).
*MdBPR6*	yes	salt sensitivity	In apple, suppression of *MdPRP6* reduces the accumulation of ROS and Na^+^ under the saline environment (Zhang et al., 2023).
*TaCCD1*	yes	alkali sensitivity	In wheat, suppression of *TaCCD1* can promote plant growth under the alkaline environment (Cui et al., 2023).

### 2.2 Feature importance analysis

**Figure 3:**
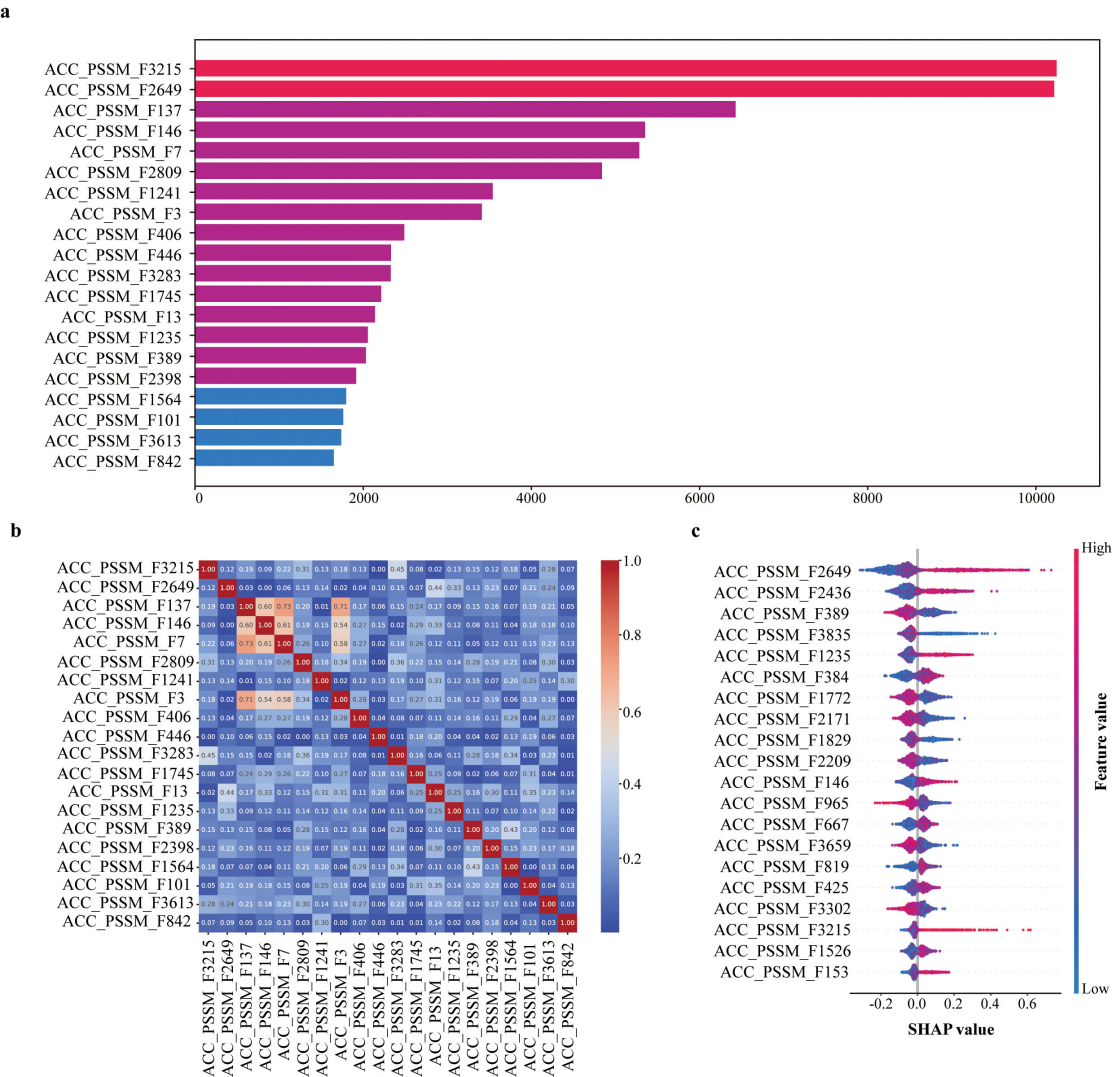
**(a)** Top 20 most important ACC_PSSM features based on gain. **(b)** Pairwise pearson correlation of top 20 ACC_PSSM features ranked by importance. **(c)** SHAP value summary for top ACC_PSSM features in SaGP.

We next analyzed the contribution of individual ACC-PSSM features to the SaGP. Based on gain values, the top 20 most important features were identified (**Figure 3a**). Features such as ACC_PSSM_F3215, ACC_PSSM_F2649, and ACC_PSSM_F137 contributed most to the model’s performance. Correlation analysis revealed low redundancy among these features, with most pairwise Pearson correlation coefficients below 0.5 (**Figure 3b**), indicating they capture distinct aspects of the input data. SHAP value analysis further confirmed the importance and directionality of these features (**Figure 3c**). For example, higher values of ACC_PSSM_F3215 and ACC_PSSM_F2649 were positively associated with model output, suggesting their strong influence in identifying tolerant genes.

To further explore their biological relevance, we investigated the potential functional significance of key features. ACC-PSSM_3215 This feature represents the autocovariance of proline residues at a lag of 9 within the PSSM (Position-Specific Scoring Matrix), capturing the evolutionary correlation between prolines separated by nine amino acid positions in the sequence. Proline is a well-established osmoprotectant in plants under salt stress, known for enhancing osmotic adjustment, stabilizing proteins and membrane structures, and mitigating oxidative damage through reactive oxygen species (ROS) scavenging. SHAP analysis revealed a positive association between higher values of this feature and the likelihood of a sequence being classified as a positive (salt-tolerant) sample. Notably, this feature exhibited significantly elevated values in salt-tolerant sequences, suggesting an enrichment of long-range proline interactions potentially involved in the formation of adaptive structural motifs or regulatory elements. These findings indicate that the model effectively captured biologically meaningful signals associated with proline-mediated stress adaptation. Importantly, despite the absence of explicit structural domain annotations, the model implicitly leveraged functional characteristics embedded within the primary sequence. The biological relevance of this proline-related feature thus provides strong support for both the predictive consistency of the positive samples and the interpretability of the model.

### 2.3 Web services of SaGP

To maximize the accessibility of the SaGP and minimize the difficulty of its use, we implemented it as a highly automated webserver (https://www.sagprediction.com/) with JavaScript, Nodejs, Tailwind CSS (responsive design), HTML5, Docker, and Nginx. The only input from the users is the protein sequences encoded by their interested genes. SaGP will automatically process the sequences and return its predictions in a formatted table. Users are allowed to download the predicted results for future use.

## 3 Discussion

Deciphering gene functions has long been the central topic in biology and bioinformatics. With the advancement of high-throughput sequencing technologies, the massive accumulation of new sequences in public databases has far exceeded the capacity of traditional wet experiments. This has led to the development of computational methods and tools to accelerate the process of gene function identification, providing guidance for wet lab experiments and reducing the costs and time associated with wet experiments. One such method is homolog-based or domain-based (e.g., BLAST), involving comparing the genomic sequences of different organisms to infer gene functions based on their similarity to known genes. Another method is machine learning to predict the functions of unknown genes based on their sequence features. Several studies have compared the performance of both methods in identifying proteins with targeted functions, such as pathogenic proteins (Li et al., 2022) and antifreeze proteins (Eslami et al., 2018; Kandaswamy et al., 2011). Overall, these studies suggest that machine learning-based methods are superior to homolog/domain-based methods regarding speed and accuracy. Consistently, in this study, we found that the performances of SaGP were higher than homolog/domain-based methods.

One possible explanation for the incapacity of homolog/domain-based methods to identify salt-alkali tolerance genes may be caused by the fast protein evolution. In plants, the main mechanisms of salt-alkali tolerance involve ions transport (e.g., Na^+^ and Ca^2+^) and detoxification (Deinlein et al., 2014; Zhang et al., 2022). Proteins with these biochemical and cellular functions tend to evolve more rapidly, resulting in low sequence similarities among homologous proteins (Devos and Valencia, 2000; Qiao et al., 2024; Xie et al., 2024) which disadvantages homolog-based and domain-based methods. Moreover, the functional space of genes/proteins is more complex than the sequence space, making it even more challenging to identify genes with specific functions based solely on sequence similarity (Devos and Valencia, 2000). SaGP, on the other hand, has the potential to overcome this challenge by capturing complex relations hidden in the sequence data based on machine learning algorithms and key protein features. Indeed, among all features, we found that ACC-PSSM performed best followed by PDT-Profile. Both ACC-PSSM and PDT-Profile capture evolutionary information (Dong et al., 2009; Liu et al., 2012). In addition, they also include sequence order effects (Dong et al., 2009; Liu et al., 2012), which may include information about local interactions/structures that are important for ion binding and transporting. Combining both groups of features barely improved model performances, suggesting that redundant information exists between them. Nevertheless, these results suggest evolution and sequence order are two crucial components for building machine learning tools to distinguish salt-alkali tolerance and non-tolerance genes in plants.

It is important to note that SaGP was trained with negative samples from *Arabidopsis thaliana*. It may have low performance to identify salt-alkali non-tolerance genes in species phylogenetically far distant from *Arabidopsis thaliana*. To evaluate the model’s generalization capability across different species, we selected three latest published genes for validation: *GhAG2* (cotton) (Yu et al., 2022), *MdBPR6* (apple) (Zhang et al., 2023), and *TaCCD1* (wheat) (Cui et al., 2023). The prediction results of SaGP were consistent with the experimental results, indicating the effectiveness of SaGP in predicting salt-alkali resistant genes across different species. The significant advancements in sequencing technologies allows us to access extensive genetic data from a variety of plants more quickly and at a lower cost. However, due to the long growth cycles and high costs, the stress tolerance genes of many plants are not well studied. The application of SaGP provides superior guidance compared to BLAST for the rapid and accurate identification of salt-alkali tolerance genes in genomic data of these plants. Additionally, to efficiently identify salt-alkali resistant genes, we developed a user-friendly and freely accessible web service platform based on SaGP. This platform allows users to obtain prediction results only by inputting protein sequences, without the need for downloading models, installing software, or deploying any environment. In summary, SaGP offers a reliable identification tool for mining novel salt-alkali tolerant genes based on large-scale data, it also can serve as a fundamental model for the development of additional automated tools, which can greatly facilitate studies in plant genetics (Li et al., 2024; Zafar et al., 2024) and crop breeding (Kumar et al., 2022; Sun et al., 2023), and promoting global agricultural sustainability (Sun et al., 2023). As the availability of genomic data continues to grow, the expansion of the training dataset will further enhance predictive capabilities of SaGP.

## 4 Materials and methods

### 4.1 Data collection and processing

#### 4.1.1 Positive samples

Saline-alkali tolerance genes were manually curated from published literature. A total of 537 experimentally validated genes from 308 gene families were collected. To reduce potential confounding factors, transcription factors were removed. Additionally, we filtered out sequences containing irregular characters (e.g., “X”) and sequences shorter than 78 amino acids—the minimum observed length among positive samples. To minimize sequence redundancy, we applied CD-HIT with a sequence identity threshold of 90%, resulting in 262 high-confidence non-redundant tolerance-related protein sequences.

#### 4.1.2 Negative samples

Negative samples were collected from the Arabidopsis thaliana genome, specifically from the TAIR database (Berardini et al., 2015), after excluding any gene families known to be associated with saline-alkali tolerance. Transcription factors and low-quality sequences (containing non-standard residues or shorter than 78 amino acids) were also removed. CD-HIT (Li et al., 2001) was used to eliminate redundant sequences at a 90% identity threshold, yielding 17,753 non-tolerance protein sequences.

To further ensure the reliability of the negative dataset, we reanalyzed RNA-seq data from Anderson et al. (2018) (Anderson et al., 2018) (GEO accession: GSE116332), which profiled gene expression in Arabidopsis thaliana under both control and salt stress conditions. Gene expression levels were quantified using StringTie, and differential expression analysis was conducted using DESeq2. Notably, none of the negative genes exhibited significant differential expression between salt-treated and control conditions, confirming their non-responsiveness to salt stress at the transcriptomic level.

### 4.2 Feature extraction and selection

**Figure 4:**
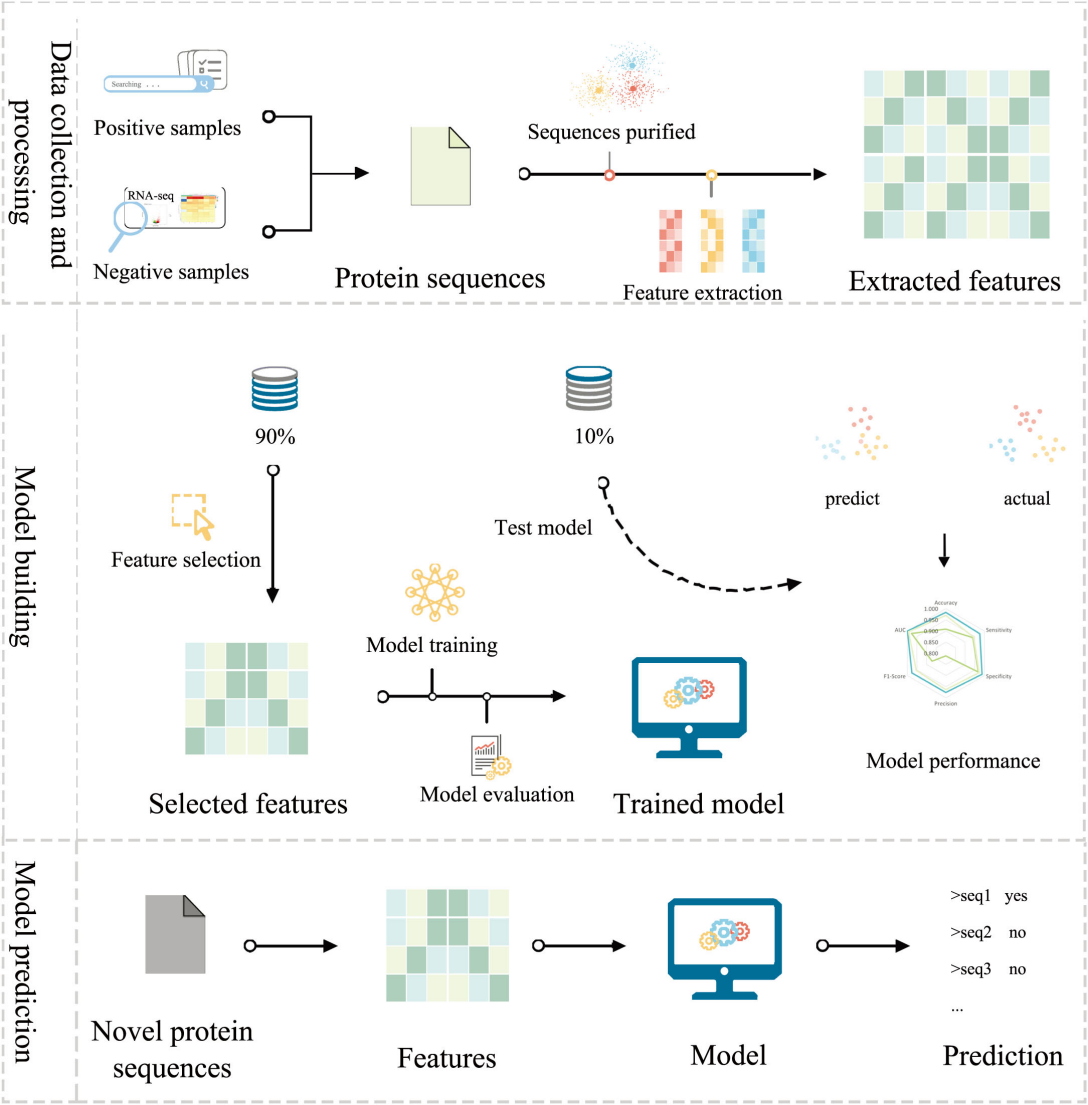
The framework of SaGP. In brief, protein sequences were used to construct SaGP. Both positive and negative sequences were validated with literature and RNA-seq data, respectively. Sequences were filtered to remove errors and redundancy. Protein features were extracted, selected, and used to train the machine learning models. The model with the best performances were evaluated based on the test dataset and were used to identify novel salt-alkali tolerance genes in plants.

Engineering protein features to capture the underlying patterns of salt-alkali tolerance and non-tolerance genes is crucial to constructing accurate SaGP models (**Figure 4**). Here, we used three programs to extract protein features, i.e., Pse-in-one2.0 (Liu et al., 2015), ftrCOOL (Amerifar et al., 2022), and MathFeature (Bonidia et al., 2022). Overall, 40 groups of protein features were extracted, representing important information about protein evolution, physicochemical properties, global and local sequence patterns, and residue interactions (**Table 3**). To reduce computational complexity and feature redundancy, features with zero variance or highly correlated with other features (absolute Pearson correlation coefficients > 0.8) were removed. The sequences data was split into training, validation, and independent test datasets with a ratio of 80:10:10. A univariate feature selection algorithm based on t-test and the training dataset was then used to select the set of features to construct machine learning models (**Figure 4**). In total, 5377 features were retained.

**Table 3:** The 40 groups of protein features extracted in our study, their abbreviations, and corresponding tools.

Features	Tools
Auto-cross covariance (ACC)	Pse-in-One 2.0
Physicochemical distance transformation (PDT)	Pse-in-One 2.0
Profile-based Auto-cross covariance (ACC-PSSM)	Pse-in-One 2.0
PseAAC of Distance-Pairs and reduced alphabet scheme (Distance Pair)	Pse-in-One 2.0
Distance-based Residue (DR)	Pse-in-One 2.0
Profile-based physicochemical distance transformation (PDT-Profile)	Pse-in-One 2.0
General parallel correlation pseudo amino acid composition (PC-PseAAC-General)	Pse-in-One 2.0
Parallel correlation pseudo amino acid composition (PC-PseAAC)	Pse-in-One 2.0
Top-n-gram	Pse-in-One 2.0
Accumulated Amino Acid Frequency (AAAF)	MathFeature
Accumulated Amino Acid Frequency with Fourier (AAAFF)	MathFeature
Electron-ion interaction potential Mapping (EIIP Mapping)	MathFeature
Integer Mapping	MathFeature
Kmer Frequency Mapping (KFM)	MathFeature
Amino acid composition (AAC)	MathFeature
Complex Networks without threshold	MathFeature
Dipeptide composition (DPC)	MathFeature
Xmer k-Spaced Ymer Composition Frequency (kGap)	MathFeature
Tripeptide composition (TPC)	MathFeature
Amino Acid to K Part Composition (AAKC)	ftrCOOL
Amino Acid Autocorrelation-Autocovariance (AAutoCor)	ftrCOOL
Amphiphilic Pseudo-Amino Acid Composition(series) (APAAC)	ftrCOOL
Adaptive skip dipeptide composition (ASDC)	ftrCOOL
Composition of k-Spaced Grouped Amino Acids pairs (CkSGAApair)	ftrCOOL
Conjoint Triad (conjointTriad)	ftrCOOL
k-Spaced Conjoint Triad (conjointTriadKS)	ftrCOOL
Composition_Transition_Distribution (CTD)	ftrCOOL
CTD Composition (CTDC)	ftrCOOL
CTD Distribution (CTDD)	ftrCOOL
CTD Transition (CTDT)	ftrCOOL
Dipeptide Deviation from Expected Mean value (DDE)	ftrCOOL
Expected Value for each Amino Acid (ExpectedValueAA)	ftrCOOL
Expected Value for Grouped Amino Acid (ExpectedValueGAA)	ftrCOOL
Expected Value for Grouped K-mer Amino Acid (ExpectedValueGKmerAA)	ftrCOOL
Expected Value for K-mer Amino Acid (ExpectedValueKmerAA)	ftrCOOL
Grouped Amino Acid K Part Composition (GAAKpartComposition)	ftrCOOL
k Grouped Amino Acid Composition (kGAAComposition)	ftrCOOL
Pseudo-Amino Acid Composition (Parallel) (PSEAAC)	ftrCOOL
Pseudo K_tuple Reduced Amino Acid Composition Type-11 (PseKRAAC_T11)	ftrCOOL
Quasi Sequence Order (QSOrder)	ftrCOOL

### 4.3 SaGP construction and evaluation

Overall, the ratio between saline-alkali tolerance and non-tolerance sequences was 1:68, which leads to an imbalanced learning problem. To address this issue and to construct SaGP with potentially optimal performance, we tested the performances of cost-sensitive methods to tackle the imbalanced learning problem (Tanha et al., 2020) (Boldini et al., 2022). compared the potentials of five cost-sensitive methods, weighted cross-entropy (WCE), Focal loss (FL), Logit-adjusted loss (LaL), Label-distribution-aware margin loss (LdaML), and Equalization loss (EL), to improve imbalanced classification in drug discovery. Here, we followed their scheme to train and evaluate our models that is Lightgbm (Ke et al., 2017) was used to train the models based on the training dataset; each model was optimized using Hyperopt (Boldini et al., 2022) based on the validation dataset; their performances were evaluated based on the independent test dataset. To assess the relative importance of different group features for identifying saline-alkali tolerance and non-tolerance genes, we evaluated each group’s features separately.

To comprehensively evaluate model performances, six metrics were calculated, i.e., Accuracy, Balanced Accuracy, F1 score, the area under the receiver operating characteristic curve (ROC-AUC), the area under the precision-recall (PR-AUC) curve and Matthew’s Correlation Coefficient (MCC). Several studies have compared the performances of these metrics for imbalanced binary classification, and in general, MCC was recommended (Chicco and Jurman, 2020, 2023). We, therefore, used MCC as the main reference to select the optimal model for SaGP.

To further confirm the power of SaGP to uncover novel saline-alkali tolerance genes, we collected three more genes from the latest publications, i.e., *GhAG2* (Yu et al., 2022), *MdBPR6* (Zhang et al., 2023), and *TaCCD1* (Cui et al., 2023), as additional tests. In addition, we assessed the performance of BLAST to identify salt-alkali tolerance genes. In brief, all salt-alkali tolerance genes in the training and validation datasets were used to construct the search database. Sequences from the test dataset were used as the query sequence of BLAST. E value 0.01 was used to indicate a significant similarity (hit).

## Data Availability

The datasets presented in this study can be found in online repositories. The names of the repository/repositories and accession number(s) can be found below: https://figshare.com/, https://figshare.com/account/home#/data.

## Supplementary material

Supplementary Figure 1 The performances of five cost-sensitive methods and 40 groups of protein features based on the test dataset. Abbreviations: EL, Equalization loss; FL, Focal loss; LaL: Logit-adjusted loss; LdaML, Label-distribution-aware margin loss; WCE, weighted cross-entropy; MCC, Matthew’s Correlation Coefficient; ROC-AUC, the area under the receiver operating characteristic curve. See **Table 3** for the details of 40 groups of protein features.
