# Supplementary figures and images for: SaGP: identifying plant saline-alkali tolerance genes based on machine learning techniques
Source: Front Plant Sci. 2025 Jul 16;16:1629794. doi: 10.3389/fpls.2025.1629794 (PMC12307364; doi:10.3389/fpls.2025.1629794)

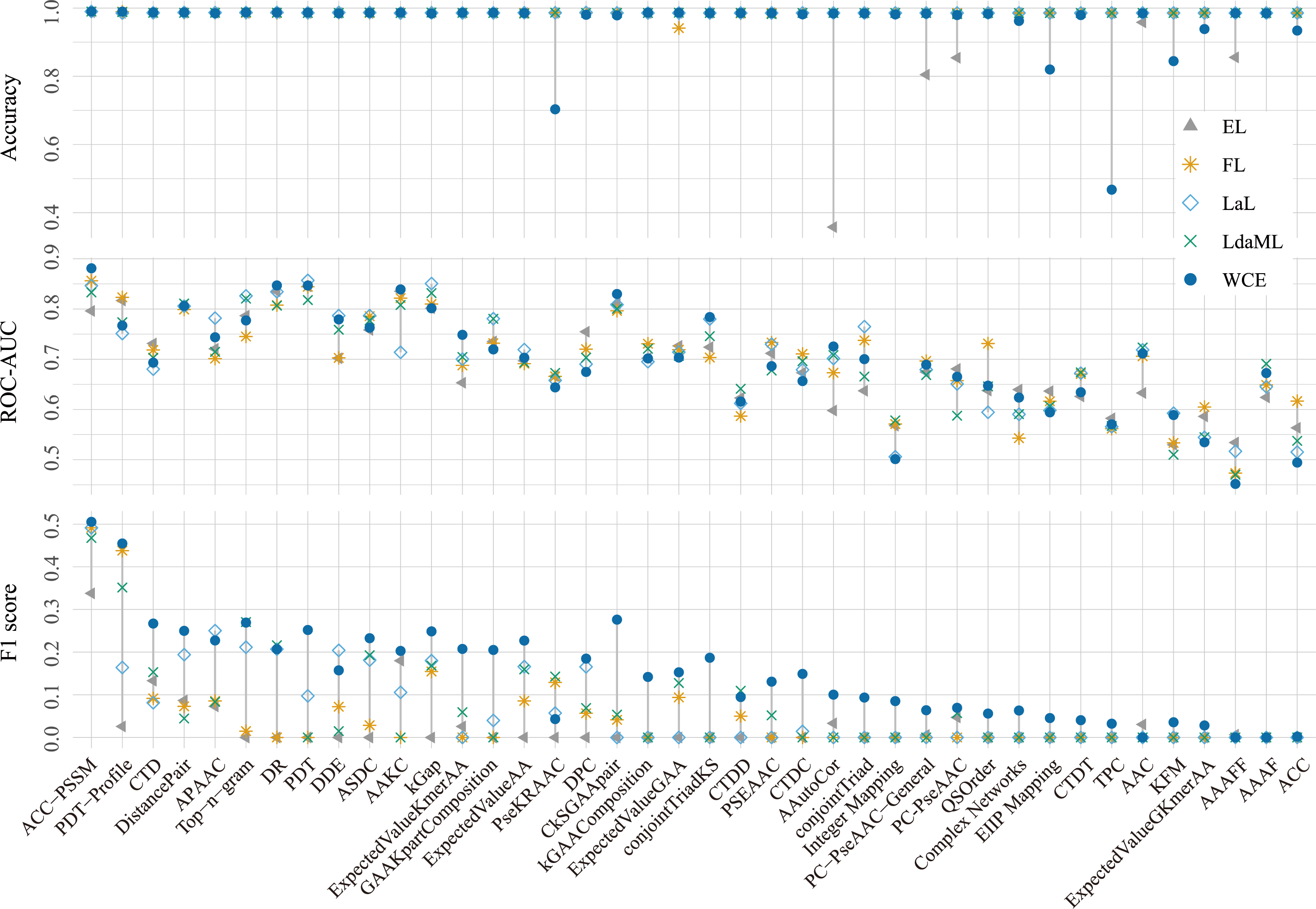

Supplement: Supplementary Figure 1 — The performances of five cost-sensitive methods and 40 groups of protein features based on the test dataset. Abbreviations: EL, Equalization loss; FL, Focal loss; LaL: Logit-adjusted loss; LdaML, Label-distribution-aware margin loss; WCE, weighted cross-entropy; MCC, Matthew’s Correlation Coefficient; ROC-AUC, the area under the receiver operating characteristic curve. See Table 3 for the details of 40 groups of protein features. [file Image1.tif]
